# Bi-allelic and tri-allelic 5-HTTLPR polymorphisms and triptan non-response in cluster headache

**DOI:** 10.1186/1129-2377-15-46

**Published:** 2014-07-21

**Authors:** Markus Schürks, Antje Frahnow, Hans-Christoph Diener, Tobias Kurth, Dieter Rosskopf, Hans-Jörgen Grabe

**Affiliations:** 1 Department of Neurology, University Hospital Essen, Hufelandstrasse 55, 45122 Essen, Germany; 2 Strausberg, Germany; 3 Inserm Research Center for Epidemiology and Biostatistics (U897), Team Neuroepidemiology, F-33000 Bordeaux, France; 4 University of Bordeaux, College of Health Sciences, F-33000 Bordeaux, France; 5 Greifswald, Germany; 6 Department of Psychiatry and Psychotherapy, University Medicine Greifswald, HELIOS Hospital Stralsund, Rostocker Chaussee 70, 18435 Stralsund, Germany

**Keywords:** 5-HTTLPR, *SLC6A4*, Polymorphism, Cluster headache, Serotonin, Triptans, Triptan response

## Abstract

**Background:**

Triptans are only effective in terminating cluster headache (CH) attacks in 70-80% of patients. Pharmacogenetic aspects of the serotonin metabolism, specifically variation in the 5-HTTLPR may be involved.

**Methods:**

Genetic association study in a well-defined cohort of 148 CH patients with information on drug response to triptans. CH was diagnosed according to the criteria of the International Headache Society. Genotypes of the 43-bp insdel (rs4795541) and A > G (rs25531) polymorphisms in the 5-HTTLPR promoter region were detected by restriction fragment length polymorphism analysis. We used logistic regression analysis to investigate the association between bi-allelic and tri-allelic genotypes and triptan non-response with genotype models.

**Results:**

Mean age at study entry among patients was 44.6 ± 10.5 years, 77.7% were men. The genotype distribution both for the bi-allelic and the tri-allelic polymorphism was in Hardy-Weinberg equilibrium. We did not find an association of the bi-allelic polymorphism with triptan non-response. While the effect estimates for the S variant of the tri-allelic polymorphisms suggested increased odds of triptan non-response in CH patients (multivariable-adjusted odds ratio [95% confidence interval]: L*L* genotype—reference; L*S* genotype—1.33 [0.53-3.32]; S*S* genotype—1.46 [0.54-3.98]), the results were not statistically significant.

**Conclusions:**

Data from our study do not indicate a role of bi-allelic and tri-allelic genotypes of the 5-HTTLPR polymorphism in triptan non-response in CH.

## Background

Cluster headache (CH) is a rare primary headache disorder characterized by excruciating unilateral periorbital pain attacks along with prominent cranial autonomic features most often occurring in bouts [1]. CH pathophysiology is incompletely understood; however, imaging studies have underlined the pivotal role of the dorsal hypothalamus [2].

Triptans are 5-hydroxytriptamine (HT, serotonin) receptor agonists targeting the 5-HT_1B/1D_ receptor subtypes with proven effectiveness in terminating acute CH attacks [3,4]. The mode of action may suggest that a lack of serotonin in the synaptic cleft or an insufficient stimulation of serotonin receptors plays an important role during CH attacks. However, only 70-80% of CH patients benefit from triptans during an acute CH attack [1,5,6], implicating pharmacogenetic reasons in the serotonin metabolism.

The main regulator of serotonin metabolism is the serotonin transporter (5-HTT), which eliminates serotonin from the synaptic cleft by transporting it from the synapses into the presynaptic neuron [7]. In humans the 5-HTT is encoded by the gene *SLC6A4*, which carries several polymorphisms in its promoter region (5-HTTLPR, 5-HTT linked polymorphic region) affecting transcription of the gene. Among those are a 43-bp insertion/deletion (insdel) polymorphism (rs4795541) [8] and a single nucleotide polymorphismus (SNP; rs25531) [9], which are located in close proximity to each other. The insdel polymorphisms yields a long (L) and a short (S) allele, the latter reducing transcriptional activity of the 5-HTT gene, thus decreasing expression and availability of the transporter [8]. The SNP rs25531 leads to an A → G exchange, which further modulates transcriptional activity. Co-occurrence of the G allele with the L allele reduces the transcriptional activity of the L allele to that of the S allele. Hence, the concept has evolved that beyond the pure bi-allelic approach to the insdel polymorphism considering the L and S alleles, from a functional point-of-view a tri-allelic approach considering the S, L_G_, and L_A_ alleles may be more appropriate.

Some recent studies have associated 5-HTTLPR polymorphisms with psychiatric disorders [10-12], addiction [13], and neurological disorders [14]. In addition, CH has been linked to psychiatric disorders [15]. Further, response to citalopram and risperidone in dementia with behavioral symptoms [16] as well as analgesic response to opioids [17] appears to be modulated by 5-HTTLPR variants.

Based on pathophysiological considerations and mode of action of triptans we investigated the association of the bi-allelic and tri-allelic 5-HTTLPR polymorphisms and triptan non-response in a cohort of well characterized CH patients.

## Methods

### Study population

Between April 2002 and March 2004 we prospectively recruited unrelated white CH patients at the Department of Neurology at the University Hospital in Essen, Germany. All study participants gave written informed consent and the local ethics committee of the University of Essen approved our study. CH diagnosis based on the criteria of the International Headache Society [18] was verified in 246 patients. Psychological tests were not performed in the patients. The design, methods of patient recruitment, and patients characteristics have been described in detail before [6]. Treatment of CH attacks was one focus of our study. We asked patients which first-line medications for CH attacks were used and which had been effective at least three times. For the present analysis we considered treatment response to triptans. In accordance with established standards, we considered treatment effective if the medication reduced CH pain by at least 50% within 15 min of a subcutaneous application or within 30 min of any other application form compared with untreated attacks [19-21]. Patients meeting these criteria were classified as "responders", those not meeting the criteria as "non-responders". Ninety-eight patients were excluded because of incomplete data on treatment response or genotyping failures, leaving data on 148 CH patients for this analysis.

### Genotype determination of the 43-bp insdel (rs4795541) and A > G (rs25531) polymorphisms in the *5-HTTLPR* promoter region

Genomic DNA was extracted from whole blood or buccal swabs using the QiaAmp Mini DNA kit (Qiagen, Hilden, Germany). We have developed a restriction fragment length polymorphism method that allows for determination of the 43-bp insdel (rs4795541) and A > G (rs25531) polymorphisms in the 5-HTTLPR promoter region within one assay. The 5-HTTLPR region was polymerase chain reaction amplified using the oligonucleotide primers SLC6A4_SE (5′- CTCCTAGGATCGCTCCTGCATC-3′) and SLC6A4_AS (5′-GGACCGCAAGGTGG- GCGGGAGGCTTGGAG-3′), resulting in amplicons of 294 base pairs for the S variant and 337 base pairs for the L variant. The restriction enzyme *BcnI* (Fermentas, St Leon-Rot, Germany) digested the rs25531 variant differentially, in addition to two constitutive restriction sites in the amplicon. This resulted in the following fragments: 200, 61, and 33 base pairs for the S allele; 243, 61, and 33 base pairs for the LA allele; and 70, 173, 61, and 33 base pairs for the LG allele. The detection of fragments of 173, 200, and 243 base pairs in 4% agarose gels allowed for allocation to the respective alleles. Representative samples of different genotypes were further verified by sequencing of the amplicons.

### Statistics

We used descriptive statistics and report mean ± standard deviation (SD) for the age at study entry as well as proportions for gender distribution and CH forms (episodic CH, chronic CH, CH of undetermined periodicity).

Considering both the 43-bp insertion/deletion (insdel) and the A > G polymorphism (rs25531) in the 5-HTTLPR promoter region the following genotypes are possible: L_A_L_A_, L_A_L_G_, L_A_S, SL_G_, SS, L_G_L_G_. For our analyses we grouped them in two different ways. First, the bi-allelic polymorphism only takes the L and S alleles into consideration, yielding the genotypes LL (L_A_L_A_, L_A_L_G_, L_G_L_G_), LS (L_A_S, SL_G_), and SS (SS). Second, the functional tri-allelic polymorphism takes into consideration that the transcriptional activity of the L allele is reduced to the level of the S allele in the presence of the variant G allele, yielding the genotypes L*L* (L_A_L_A_), L*S* (L_A_L_G_, L_A_S), S*S* (SS, SL_G_, L_G_L_G_).

Genotype and allele frequencies between responders and non-responders were compared using chi-square test statistic. We also tested for Hardy-Weinberg Equilibrium (HWE).

We used logistic regression models to evaluate the association between bi-allelic and tri-allelic 5-HTTLPR genotypes and non-response to triptans. We used an approach free of assumptions and built genotype models (2df). These models allow for independent comparison of the heterozygous and homozygous variant genotypes to the reference genotype. We considered the LL (bi-allelic polymorphism) and L*L* (tri-allelic polymorphism) genotypes as reference. We calculated crude and multivariable-adjusted odds ratios (ORs) and 95% confidence intervals (CIs). For the multivariable-adjusted model we *a priori* considered age at study entry (linear), gender (men, women), and CH form (episodic CH, chronic CH, CH of undetermined periodicity) as covariates.

We also investigated for a potential linear effect of the variant alleles across the genotypes on triptan non-response using a test for trend.

All analyses were performed using SAS version 9.1 (SAS Institute Inc, Cary, NC). All p-values were two-tailed, and we considered a p-value < 0.01 as significant [22].

## Results

Table 1 summarizes the baseline characteristics of triptan users overall and according to the 5-HTTLPR genotypes. The mean age at study entry among all 148 triptan users was 44.6 ± 10.5 years, 77.7% were men. The majority had episodic CH (69.6%), 18.9% had chronic CH, and in 11.5% periodicity was undetermined. These characteristics did not differ across genotypes for either of the two polymorphisms investigated. Of the 148 triptan users 46 (31.1%) were triptan non-responders.

**Table 1:** Baseline characteristics of triptan users overall and according to 5-HTTLPR polymorphism

	**All triptan users (n = 148)**	**Bi-allelic 5-HTTLPR polymorphism**			**p-value†**	**Functional tri-allelic 5-HTTLPR polymorphism**			**p-value†**
		**LL (n = 44)**	**LS (n = 70)**	**SS (n = 34)**		L*L* (n = 37)	L*S* (n = 70)	S*S* (n = 41)	
Age (mean ± SD), years	44.6 ± 10.5	45.5 ± 12.0	43.9 ± 9.7	44.9 ± 10.3	0.82	45.5 ± 12.0	44.5 ± 10.1	44.0 ± 10.0	0.82
Men (%)	115 (77.7)	35 (79.6)	53 (75.7)	27 (79.4)	0.86	29 (78.4)	55 (78.6)	31 (75.6)	0.93
CH form,%									
Episodic	103 (69.6)	27 (61.4)	52 (74.3)	24 (70.6)	0.47	22 (59.5)	50 (71.4)	31 (75.6)	0.28
Chronic	28 (18.9)	10 (22.7)	13 (18.6)	5 (14.7)		8 (21.6)	15 (21.4)	5 (12.2)	
Undetermined periodicity	17 (11.5)	7 (15.9)	5 (7.1)	5 (14.7)		7 (18.9)	5 (7.1)	5 (12.2)	

5-HTTLPR, 5-hydroxytryptamine (=serotonin) transporter linked polymorphic region; CH, cluster headache.

†p-value from chi-square test for categorical variables and from Kruskal Wallis test for linear variables.

The observed genotype distributions for the bi-allelic and tri-allelic 5-HTTLPR polymorphisms were in HWE among non-responders and responders (Table 2). There was no significant difference in the genotype and allele distribution for either of the polymorphisms between non-responders and responders (Table 2).

**Table 2:** Genotype and allele distribution of the 5-HTTLPR polymorphisms among triptan responders (n = 102) and non-responders (n = 46)

**Response status**	**Bi-allelic**							
	**HWE**	**Genotype frequencies**			**p-value**	**Allele frequencies**		**p-value**
		**LL (44)**	**LS (70)**	**SS (34)**		**L (158)**	**S (138)**	
Responders	1.00	28.3 (29)	50.0 (51)	21.6 (22)	0.61	0.53 (109)	0.47 (95)	0.98
Non-responders	0.25	32.6 (15)	41.3 (19)	26.1 (12)		0.53 (49)	0.47 (43)	
	**Tri-allelic**							
	**HWE**	**Genotype frequencies**			**p-value**	**Allele frequencies**		**p-value**
		L*L* (37)	L*S* (70)	S*S* (41)		L* (144)	S* (152)	
Responders	0.56	26.5 (27)	47.1 (48)	26.5 (27)	0.79	0.50 (102)	0.50 (102)	0.49
Non-responders	0.78	21.7 (10)	47.8 (22)	30.4 (14)		0.46 (42)	0.54 (50)	

HWE: p-value for Hardy-Weinberg equilibrium from exact test.

Genotype frequencies are in%, allele frequencies in decimals. Numbers are in parentheses.

The association between the 5-HTTLPR genotypes and triptan non-response is summarized in Table 3. We did not find an association with triptan non-response for genotypes of the bi-allelic polymorphism (multivariable-adjusted OR [95% CI]: LS vs. LL—0.75 [0.32-1.73], p = 0.40; SS vs. LL—1.04 [0.40-2.72], p = 0.66). While the effect estimates provided some suggestion that the S* allele of the functional tri-allelic polymorphism confers increased risk for non-response to triptans, these results were not statistically significant (multivariable-adjusted OR [95% CI]: L*S* vs. L*L*—1.33 [0.53-3.32], p = 0.79; S*S* vs. L*L*—1.46 [0.54-3.98], p = 0.56). In addition, the test for trend did not suggest a linear effect of the variant alleles across the genotypes on triptan non-response for either polymorphism.

**Table 3:** Association between the 5-HTTLPR genotypes and triptan response in CH

**5-HTTLPR genotype**	**Crude model**			**Multivariable-adjusted model†**		
**Bi-allelic**	**OR (95% CI)**	**p-value**	**p**_**trend**_	**OR (95% CI)**	**p-value**	**p**_**trend**_
LL	Referent	----	0.98	Referent	----	0.98
LS	0.72 (0.32-1.63)	0.32		0.75 (0.32-1.73)	0.40	
SS	1.06 (0.41-2.70)	0.60		1.04 (0.40-2.72)	0.66	
**Functional tri-allelic**						
L*L*	Referent	----	0.50	Referent		0.46
L*S*	1.24 (0.51-3.00)	0.90		1.33 (0.53-3.32)	0.79	
S*S*	1.40 (0.53-3.70)	0.56		1.46 (0.54-3.98)	0.56	

5-HTTLPR, 5-hydroxytryptamine (=serotonin) transporter linked polymorphic region; CH, cluster headache; OR, odds ratio; CI, confidence interval.

OR are derived from genotype models (2df).

†adjusted for age, gender, and CH form.

## Discussion

The results of our study do not indicate an association between genotypes of the bi-allelic or tri-allelic 5-HTTLPR polymorphisms and triptan non-response among patients with CH. While the effect estimates from models of the tri-allelic variant suggest an increased risk for a non-response to triptans among CH patients carrying the S allele, the results were not statistically significant.

Medication non-response in CH is a phenomenon occurring in about 20-30% of patients and applying to both acute and preventive treatment [1,5,6]. Especially non-response to triptans, which are the most effective and reliable drugs for attack abortion, is worrisome since this may leave the patient without help during an attack. While timing of the medication application and environmental factors can influence treatment success, pharmacogenetic reasons may play a role. One previous study did not find an association of genetic variation in the hypocretin receptor 2 with treatment response to acute and preventive CH medication [23]. Although an association of the gene variant with disease phenotype has been reported [24,25], the functionality of the gene variant has not yet been demonstrated [26]. However, the C825T polymorphism in the gene coding for the G protein β3 subunit (GNB3) has been shown to modulate treatment response to triptans [27]. While serotonin receptors that couple to G proteins are the target for both the natural ligand serotonin and for triptans [28], there is doubt about their pharmacogenetic role in triptan response [29].

Based on the central role of serotonin in the pathophysiology of CH and these previous findings, it is plausible to hypothesize that further genetic variants may impact pharmacological effects involving the serotonin system and thus triptan response. The main regulator of serotonin metabolism and serotonin concentration in the synaptic cleft is the serotonin transporter (5-HTT) [7] coded by the gene SLC6A4. Variants in the promoter region of this gene termed 5-HTTLPR have previously been investigated with respect to other headache disorders including migraine [30] and medication-overuse headache [31]; however, not with regard to CH.

The effect estimates from our models of the tri-allelic 5-HTTLPR polymorphism show a non-significant increased risk for triptan non-response among CH patients carrying the S allele. This might suggests that physiological clearing of serotonin from the synaptic cleft is a prerequisite for triptan response. In such a scenario physiological levels of serotonin in the synaptic cleft would be secured by presence of the L_A_ allele, thus allowing for an adequate triptan response. In contrast, presence of the L_G_/S allele would result in reduced number of functionally intact 5-HTT thus elevating the serotonin concentration above the "normal" obviating adequate triptan response. However, this hypothesis needs to be further investigated in future studies.

The strength of our study lies in the well-characterized cohort of CH patients with diagnosis verified based on International Headache Society criteria [18]. Further, some limitations should be considered. First, the sample size of our study may be limited to detect small to moderate effects of the 5-HTTLPR polymorphism on triptan non-response in CH. In particular, the number of patients not responding to triptans was limited (n = 46). However, our results allow the conclusion that the 5-HTTLPR does not have a large effect on triptan non-response. Second, all participants were of Caucasian origin, thus generalizability to other populations may be limited. Finally, we were unable to investigate potential gene-gene and gene-environment interactions due to reduced power, which may be important for triptan non-response.

## Conclusion

The results of our study do not indicate an association between genotypes of the 5-HTTLPR polymorphisms and triptan non-response in CH. Findings from the tri-allelic 5-HTTLPR polymorphism analysis may be hypothesis generating. Specifically, investigating the association between this variant and triptan non-response in CH in independent studies with carefully characterized patients according to International Headache Society criteria appears promising. While it is notoriously difficult to gather a sufficiently large number of CH with adequate information on triptan response due to the low prevalence of the disorder, such an approach should still be considered considering the option of data pooling later.

## Abbreviations

5-HT: 5-hydroxytryptamine = serotonin; 5-HTT: Serotonin transporter; 5-HTTLPR: 5-HTT linked polymorphic region, located in the promoter region of the SLC6A4 gene; bp: Base pairs; CH: Cluster headache; CI: Confidence interval; HWE: Hardy-Weinberg Equilibrium; Insdel: Insertion/deletion polymorphism; OR: Odds ratio; SD: Standard deviation; SLC6A4: Gene coding for the serotonin transporter; SNP: Single nucleotide polymorphism.

## Competing interests

None of the full disclosures listed constitute a competing interest with respect to the particular matter of the manuscript.

## Authors’ contributions

MS acquired the data, designed the study, performed the statistical analysis, interpreted the data, and drafted the manuscript. AF acquired the data, performed the genotyping, and critically revised the manuscript. HCD acquired the data and critically revised the manuscript. TK contributed to the study design and critically revised the manuscript. DR acquired the data, contributed to the study design, and critically revised the manuscript. HJG contributed to the study design, interpreted the data, and critically revised the manuscript. All authors read and approved the final manuscript.
